# Predictors for development of complete and incomplete intestinal metaplasia (IM) associated with *H*. *pylori* infection: A large-scale study from low prevalence area of gastric cancer (IM-HP trial)

**DOI:** 10.1371/journal.pone.0239434

**Published:** 2020-10-01

**Authors:** Natsuda Aumpan, Ratha-Korn Vilaichone, Pongjarat Nunanan, Soonthorn Chonprasertsuk, Sith Siramolpiwat, Patommatat Bhanthumkomol, Bubpha Pornthisarn, Tomohisa Uchida, Virunpat Vilaichone, Arti Wongcha-Um, Yoshio Yamaoka, Varocha Mahachai

**Affiliations:** 1 Department of Medicine, Gastroenterology Unit, Faculty of Medicine, Thammasat University Hospital, Pathumthani, Thailand; 2 Department of Medicine, Chulabhorn International College of Medicine (CICM) at Thammasat University, Pathumthani, Thailand; 3 Digestive Diseases Research Center (DRC), Thammasat University Hospital, Pathumthani, Thailand; 4 Department of Molecular Pathology, Oita University Faculty of Medicine, Yufu, Japan; 5 Department of Environmental and Preventive Medicine, Oita University Faculty of Medicine, Yufu, Japan; 6 Gastrointestinal and Liver Center, Bangkok Medical Center, Bangkok, Thailand; Istituto di Ricovero e Cura a Carattere Scientifico Centro di Riferimento Oncologico della Basilicata, ITALY

## Abstract

**Background:**

Gastric intestinal metaplasia (IM) is precancerous lesion of gastric cancer related to *H*. *pylori* infection. There has been limited data about IM and associated risk factors. This study aimed to determine risk factors related to development of IM to guide proper management.

**Methods:**

1,370 patients undergoing UGI endoscopy at Thammasat University Hospital, Thailand were included between January 2018-August 2019. Patients’ data including baseline characteristics, laboratory results, and histopathology from medical database were extensively reviewed. Immunohistochemical staining for p53 expression from gastric biopsies was also performed.

**Results:**

Overall *H*. *pylori* prevalence was 43.8%. Mean age was 60.7 years and 45% of whom were males. Chronic gastritis was observed in 1,064(77.7%) patients, while 223(16.3%) had IM. Of 223 patients with IM, 194(87%) patients had complete IM, while 29 (13%) had incomplete IM. In groups of complete and incomplete IM, current *H*. *pylori* infection rates were 66.5% and 58.6%, respectively. The BMI of incomplete IM group(27.4) was significantly higher than BMI of complete IM group (23.6). Overweight and obese patients (BMI ≥23 kg/m^2^) were significantly associated with higher risk for the development of incomplete IM (OR 3.25; 95%CI 1.14–9.27, p = 0.027). Males, age >50 years, and current *H*. *pylori* infection were significantly higher in IM than chronic gastritis group with OR 1.43 (95%CI 1.01–2.03, p = 0.048), OR 1.67 (95% CI 1.08–2.57, p = 0.021), and OR 3.14 (95% CI 2.29–4.30, p<0.001), respectively. During 20 months of study, there were 15 patients (1.1%) diagnosed with gastric cancer and 1-year survival rate was only 60%.

**Conclusions:**

Males, age >50 years, and current *H*. *pylori* infection are significant predictors for the presence of intestinal metaplasia. BMI might be beneficial for using as a predictive risk factor to reduce the development of incomplete intestinal metaplasia. *H*. *pylori* eradication could be an effective way to prevent the development of gastric precancerous lesions.

## Introduction

Gastric cancer is the third leading cause of global cancer-related mortality. In 2018, almost 800,000 lives were lost and over 1 million new cases were newly diagnosed with gastric cancer [1]. The prevalence of gastric cancer was particularly high in Asia exceeding three-quarters of all patients worldwide. In contrast, low prevalence of gastric cancer was reported in North America, Southeast Asia, and Africa [2]. Lauren classification categorizes gastric adenocarcinomas into two histologic types, intestinal and diffuse type. The latter is more aggressive and commonly diagnosed at an advanced stage [3]. The diffuse-type gastric carcinogenesis prominently occurs through molecular defect involving in cellular adhesion, whereas the intestinal type depends on interactions among host genetics, environment and pathogen resulting in multistep precancerous cascades [4, 5]. However, both types are associated with inflammation triggered by *Helicobacter pylori (H*. *pylori)* infection, the strongest risk factor for gastric cancer [6].

Gastric intestinal metaplasia (IM) is an intermediate precancerous lesion of intestinal-type gastric cancer. *H*. *pylori* initiates carcinogenesis through chronic inflammatory response in the gastric mucosa. Persistent inflammation causes atrophic gastritis or loss of gastric glands which are subsequently replaced by intestinal epithelium [7]. Considered as “the point of no return”, IM has potential to progress to dysplasia and eventually becomes cancer cells [8, 9]. IM imposes 1.8% higher risk of gastric cancer in 10-year follow-up [10]. The prevalence of IM varies among regions, ranging from 3.4% in North Europe to 23.9% in South America [11]. Certain ethnic groups such as Hispanic and East Asian have significantly higher IM and gastric cancer prevalence than others [12]. Apart from ethnicities, genetic mutations including amplification of oncogenes and inactivation of tumor suppressor genes are also associated with development of gastric cancer. p53 gene mutation is one of altered tumor suppressor genes detected in both early or advanced gastric adenocarcinomas [13]. However, there were few studies about p53 and its association with IM.

So far, there has been limited data about IM prevalence and associated risk factors in Thailand, a country with low prevalence of gastric cancer. This study aimed to determine risk factors related to the development of IM and evaluate p53 expression in patients with gastric IM.

## Materials and methods

### Study design

This retrospective cohort study was conducted at Thammasat University Hospital between January 2018 and August 2019. The inclusion criteria were Thai patients aged more than 15 years old who underwent upper gastrointestinal endoscopy as indicated for diagnostic evaluation of symptoms (e.g., dyspepsia, upper gastrointestinal bleeding, unexplained weight loss) [14]. All patients in this study were diagnosed with chronic gastritis, precancerous lesion, or adenocarcinoma on pathology report of gastric biopsies. Patients undergoing endoscopic procedure without gastric mucosal biopsy were excluded from this study. Demographic data, clinical presentation, laboratory results including a complete blood count and a comprehensive metabolic panel, endoscopic and histopathological findings, current *H*. *pylori* infection status, and treatment outcomes were extracted from medical database and extensively reviewed.

The primary aims were risk factors associated with the development of intestinal metaplasia including complete and incomplete type. The secondary aim was to evaluate p53 expression in patients with gastric IM.

### Routine protocol for the endoscopic mapping

Upper gastrointestinal endoscopy was performed in each patient with adequate mucosal visualization enhanced by air insufflation, mucosal cleaning technique, and sufficient examination time at least 7 minutes from intubation to extubation [15]. Photographic documentation of gastric areas including antrum, pylorus, incisura, lesser curvature, greater curvature, fundus, and cardia was obtained during upper endoscopy. For gastric mapping in this study, a minimum of 3 non-targeted biopsies were obtained from antrum, corpus, and incisura, along with at least two biopsies from any macroscopic targeted lesion. A biopsy from antrum was sent for rapid urease test, whereas biopsies from both antrum and body were sent for histopathology.

### Histopathology

Hematoxylin and eosin (H&E) staining was used in the histopathological diagnosis. Giemsa staining was used as a special staining method for the diagnosis of *H*. *pylori* infection. The diagnosis of gastric precancerous lesions and current *H*. *pylori* infection were based on histopathological features as follows.

**Current *H*. *pylori* infection** was defined as histological identification of *H*. *pylori*, curved rod-shaped bacteria, and/or the positive rapid urease test.

**Precancerous lesion** was defined as any gastric mucosa pathology of atrophic gastritis, intestinal metaplasia, or dysplasia.

**Chronic gastritis** was defined as more than 2 to 5 of mononuclear leukocytes including lymphocytes, plasma cells, and macrophages per high power field (x40 objective) in the lamina propria of gastric mucosa pathology [16]. This study included all causes of chronic gastritis such as *H*. *pylori*-associated gastritis, drug-induced gastritis, and bile reflux gastritis.

**Atrophic gastritis** was defined as loss of gastric glandular cells resulting from chronic inflammation of gastric mucosa. Atrophic gastritis can be classified into two subtypes by etiologies. The first one was autoimmune metaplastic atrophic gastritis (AMAG) causing corpus-predominant atrophy by immune-mediated destruction of parietal cells leading to loss of intrinsic factor and reduced acid production. The other one was environmental metaplastic atrophic gastritis (EMAG) most commonly caused by *H*. *pylori* infection. The latter could result in antral-predominant atrophy or multifocal atrophic areas as the disease progressed [17].

**Intestinal metaplasia (IM)** was defined as the replacement of gastric oxyntic or antral mucosa by intestinal epithelium. IM was classified as complete or incomplete type by histopathologic difference of H&E staining according to Filipe and Jass [18]. Complete IM comprises straight crypts lined by small intestinal epithelium with a well-defined brush border and mature goblet cells, whereas incomplete IM is composed of architecturally-distorted crypts lined by colonic epithelium with multiple intracytoplasmic mucin droplets varying in size and shape without a brush border [18, 19].

**Dysplasia** was defined as gastric epithelium composed of enlarged, hyperchromatic, and disorganized nuclei but still confined within the basement membrane. Dysplasia could ultimately transform to invasive adenocarcinoma at a rate of 0.6 to 6% in a 5-year follow-up [10].

### Immunohistochemical analysis of p53 expression

Paraffin-embedded blocks of gastric tissue containing intestinal metaplasia were retrieved from Department of Pathology, Thammasat university hospital. Four-micron sections of each patient’s tissue specimens were cut by microtome, floated out on warm water, and subsequently mounted on microscope slides. These tissue specimens were then immunostained for the presence of p53 protein using anti-p53 antibody (DO-5, Leica Biosystems). These monoclonal antibodies can recognize epitopes of both mutant and wild type p53 protein. Tissue sections were later incubated in peroxidase blocking solution for 10 minutes at room temperature. Immune complexes were detected by chromogen [20]. The protocol for p53 immunochemistry is available at http://dx.doi.org/10.17504/protocols.io.bigjkbun. A specimen of hepatocellular carcinoma with a known p53 expression served as a positive control, while normal gastric mucosa was used as a negative control. The p53 positive cell was defined by brown nuclear staining of the epithelial cell. The p53 immunohistochemistry score was graded from 0 to 5 according to percentage of positive cells as follows: 0 (0%), 1 (<10%), 2 (10–25%), 3 (26–50%), 4 (51–75%), and 5 (>75%). Positive p53 staining was defined as a score of ≥2.

### Statistical analysis

All data were analysed by using SPSS version 22 (SPSS Inc., Chicago, IL, USA). The demographic data were analysed by Fisher’s exact test, or Chi-square test where appropriate. Statistical significance was defined as p-value of less than 0.05. The study received ethical approval by the Human Research Ethics Committee of Thammasat University, Thailand and was conducted according to the good clinical practice guideline, as well as the Declaration of Helsinki. All data had been fully anonymized before they were accessed. The Ethics Committee waived the requirement for informed consent because of no greater than minimal risk for participants. The project number of ethical approval was MTU-EC-IM-6-160/62.

## Results

**Fig 1:**
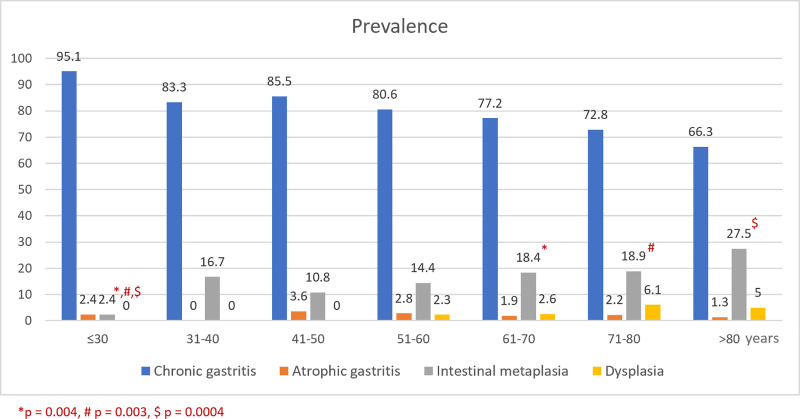
Distribution of chronic gastritis and precancerous lesions classified by age group (N = 1,355).

**Fig 2:**
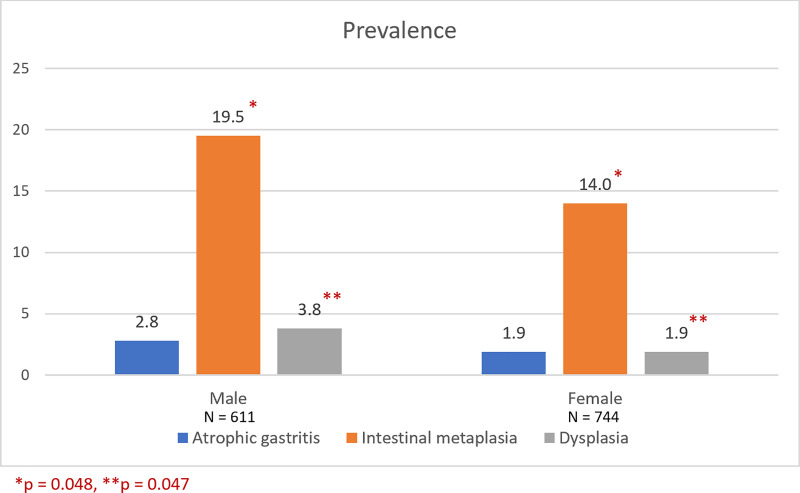
Prevalence of precancerous lesions classified by gender (N = 1,355).

Total of 1,370 Thai patients were included in the study. There were 617 men and 753 women with the mean age of 60.7 ± 13.3 (range 16–96) years. All patients had indications for upper gastrointestinal endoscopy suggested by Gastroenterology Association of Thailand (GAT). The indications were dyspepsia (58.3%), gastrointestinal bleeding (12.3%), anemia (9.5%), weight loss (6.3%), dysphagia (2.7%), and others (e.g. chronic abdominal pain, suspected gastrointestinal malignancy-associated thrombosis) (10.9%). Patients were classified according to their pathological findings of gastric mucosa. The prevalence of chronic gastritis was 77.7%, followed by intestinal metaplasia (16.3%). However, atrophic gastritis and dysplasia were infrequently found at approximately 2%. Gastric precancerous lesions were predominantly present in men. The mean age of patients having intestinal metaplasia or dysplasia was higher than other groups as demonstrated in Table 1. Atrophic gastritis, intestinal metaplasia and dysplasia were detected at the minimum age of 16, 30, and 51 years, respectively. The prevalence of intestinal metaplasia and dysplasia increased with age when categorized by age group. Men were significantly related to higher prevalence of IM and dysplasia than women with OR 1.43 (95%CI 1.01–2.03, p = 0.048) and OR 2.00 (95%CI 1.01–3.97, p = 0.047), respectively. Distribution of precancerous lesions classified by age group and gender was shown in Figs 1 and 2, respectively. The overall prevalence of *H*. *pylori* infection was 43.8%.

**Table 1:** Baseline characteristics of study population.

Factors	Total		Chronic gastritis		Atrophic gastritis		Intestinal metaplasia		Dysplasia		Gastric cancer	
Total patients	1,370		1,064 (77.7%)		31 (2.3%)		223 (16.3%)		37 (2.7%)		15 (1.1%)	
Male (%)	617 (45.0%)		452 (42.5%)		17 (54.8%)		119 (53.4%)		23 (62.2%)		6 (40.0%)	
Mean age ± SD	60.7 ± 13.3		59.8 ± 13.5		59.5 ± 13.7		64.2 ± 12.6		68.7 ± 9.9		59.5 ± 7.8	
Range (years)	16–96		20–94		16–86		30–96		51–88		42–68	
*H*. *pylori* infection	600 (43.8%)		400 (37.6%)		20 (64.5%)		146 (65.5%)		27 (73.0%)		7 (46.7%)	
Comorbidities												
None	317	(23.1%)	241	(22.7%)	11	(35.5%)	53	(23.8%)	5	(13.5%)	7	(46.7%)
Diabetes mellitus	275	(20.1%)	209	(19.6%)	9	(29.0%)	45	(20.2%)	9	(24.3%)	3	(20.0%)
Hypertension	549	(40.1%)	416	(39.1%)	10	(32.3%)	101	(45.3%)	18	(48.6%)	4	(26.7%)
Dyslipidemia	506	(36.9%)	395	(37.1%)	11	(35.5%)	78	(35.0%)	17	(45.9%)	5	(33.3%)
Family history of gastric cancer	12	(0.9%)	8	(0.8%)	0	(0%)	3	(1.3%)	1	(2.7%)	0	(0%)
Smoking (%)	172	(13.7%)	120	(12.4%)	2	(8.3%)	37	(17.5%)	11	(30.6%)	2	(14.3%)
Alcohol (%)	236	(18.8%)	166	(17.2%)	4	(16.7%)	51	(24.1%)	13	(35.1%)	2	(14.3%)

Patients with chronic gastritis (1,064 patients) and intestinal metaplasia (223 patients) were included for both univariate and multivariate analysis. Males, age more than 50 years, and current *H*. *pylori* infection were risk factors significantly associated with intestinal metaplasia compared to chronic gastritis group with OR 1.43 (95%CI 1.01–2.03, p = 0.048), OR 1.67 (95% CI 1.08–2.57, p = 0.021), and OR 3.14 (95% CI 2.29–4.30, p <0.001), respectively. However, alcohol use appeared to be factors related to intestinal metaplasia in the univariate analysis but could not reach statistical significance in the multivariate analysis. Body mass index (BMI), comorbidities, family history of gastric cancer, smoking status, and endoscopic findings were not different between groups. Univariate and multivariate analysis of associated risk factors in patients with chronic gastritis and intestinal metaplasia was demonstrated in Tables 2 and 3.

**Table 2:** Univariate analysis of associated risk factors and odds ratio in patients with chronic gastritis and intestinal metaplasia.

Risk factors	Chronic gastritis		Intestinal metaplasia		Odds ratio (95% CI)		P-value
	**(**N **=** 1,064**)**		**(**N **=** 223**)**				
**Male (%)**	**452**	**(42.5%)**	**119**	**(53.4%)**	**1.55**	**(1.16–2.07)**	**0.003**
**Age (years ± SD)**	**59.8 ± 13.5**		**64.2 ± 12.6**		**-**		**<0.001**
**>50 years**	**833**	**(78.3%)**	**194**	**(87.0%)**	**1.86**	**(1.22–2.81)**	**0.004**
BMI (kg/m^2^ ± SD)	23.8 ± 4.3		24.2 ± 5.3		-		0.379
***H*. *pylori* infection (%)**	**400**	**(37.6%)**	**146**	**(65.5%)**	**3.15**	**(2.33–4.26)**	**<0.001**
Underlying diseases							
None	241	(22.7%)	53	(23.8%)	1.07	(0.76–1.50)	0.718
Diabetes mellitus	209	(19.6%)	45	(20.2%)	1.03	(0.72–1.48)	0.855
Hypertension	416	(39.1%)	101	(45.3%)	1.29	(0.96–1.73)	0.087
Dyslipidemia	395	(37.1%)	78	(35.0%)	0.91	(0.67–1.23)	0.546
Chronic kidney disease	68	(6.4%)	16	(7.2%)	1.13	(0.64–1.99)	0.667
Cardiovascular disease	84	(7.9%)	23	(10.3%)	1.34	(0.83–2.18)	0.236
Cirrhosis and hepatitis	186	(17.5%)	38	(17.0%)	0.97	(0.66–1.42)	0.875
Pulmonary diseases	30	(2.8%)	8	(3.6%)	1.28	(0.58–2.84)	0.539
Neurological disorders	60	(5.6%)	20	(9.0%)	1.65	(0.97–2.80)	0.064
Rheumatic diseases	66	(6.2%)	15	(6.7%)	1.09	(0.61–1.95)	0.770
Malignancy	93	(8.7%)	20	(9.0%)	1.03	(0.62–1.71)	0.913
Family history of gastric cancer (%)	8	(0.8%)	3	(1.3%)	1.80	(0.47–6.84)	0.388
Smoking (%)	120	(12.4%)	37	(17.5%)	1.49	(1.00–2.23)	0.052
**Alcohol (%)**	**166**	**(17.2%)**	**51**	**(24.1%)**	**1.53**	**(1.07–2.18)**	**0.020**

**Table 3:** Multivariate analysis of associated risk factors and odds ratio in patients with chronic gastritis and intestinal metaplasia.

Risk factors	Odds ratio (95% CI)		p-value
**Male**	**1.43**	**(1.01–2.03)**	**0.048**
**Age >50 years**	**1.67**	**(1.08–2.57)**	**0.021**
**Current *H*. *pylori* infection**	**3.14**	**(2.29–4.30)**	**<0.001**
Alcohol	1.18	(0.77–1.80)	0.443
Family history of gastric cancer	2.72	(0.60–12.33)	0.194

**Fig 3:**
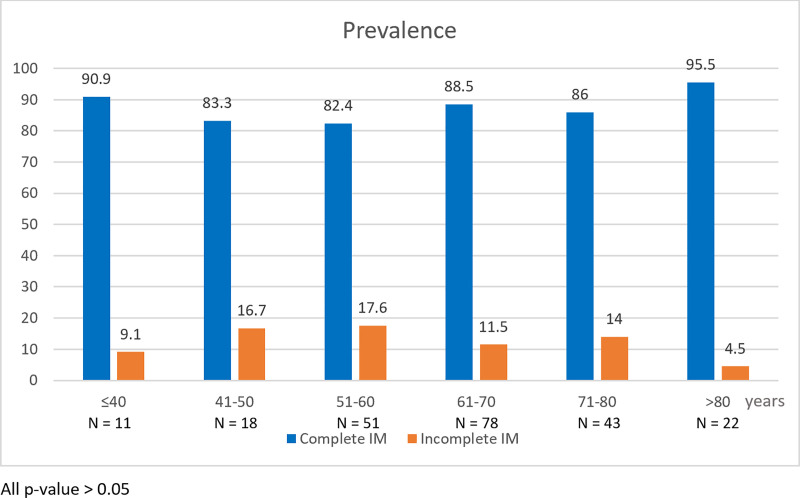
Prevalence of complete and incomplete IM classified by age group (N = 223).

Two hundred and twenty-three patients with IM were divided into two groups. Of all patients with IM, 194 (87%) patients had complete IM, while 29 (13%) had incomplete IM. The prevalence of incomplete IM increased with age and almost doubled in the age group of 41–50 (OR 2.0; 95%CI 0.18–22.06, p = 0.571) and 51–60 years (OR 2.14; 95%CI 0.24–18.92, p = 0.493) compared to the group of ≤40 years. The prevalence of IM types classified by age group was demonstrated in Fig 3. Both IM types displayed male predominance. Patients with complete and incomplete IM had the mean age of 64.6 and 62.1 years, respectively. The current *H*. *pylori* infection rate of patients with incomplete IM (58.6%) was slightly lower than the complete IM group (66.5%) (OR 0.71; 95%CI 0.32–1.58, p = 0.407). The mean body mass index (BMI) of incomplete IM group (27.4) was significantly higher than the BMI of complete IM (23.6). Overweight defined by Asian BMI criteria (BMI ≥ 23 kg/m^2^) was significantly associated with incomplete IM (OR 3.25; 95%CI 1.14–9.27, p = 0.027). There was no difference of laboratory result including complete blood count, plasma glucose, tumor markers (CEA and CA19-9) between complete and incomplete IM groups. Univariate and multivariate analysis of associated risk factors in patients with complete and incomplete IM was demonstrated in Tables 4 and 5.

**Table 4:** Univariate analysis of associated risk factors and odds ratio in patients with complete and incomplete IM.

Risk factors	Complete IM		Incomplete IM		Odds ratio (95% CI)		P-value
	(N = 194)		(N = 29)				
Male (%)	103	(53.1%)	16	(55.2%)	1.09	(0.50–2.38)	0.834
Age (years ± SD)	64.6 ± 12.7		62.1 ± 11.6		-		0.329
>50 years	169	(87.1%)	25	(86.2%)	0.93	(0.30–2.88)	0.892
**BMI (kg/m**^**2**^ **± SD)**	**23.6 ± 4.6**		**27.4 ± 7.6**		**-**		**0.029**
**BMI ≥23 kg/m**^**2**^	**75**	**(54.0%)**	**19**	**(79.2%)**	**3.24**	**(1.15–9.18)**	**0.027**
*H*. *pylori* infection (%)	129	(66.5%)	17	(58.6%)	0.71	(0.32–1.58)	0.407
Underlying diseases							
None	47	(24.2%)	6	(20.7%)	0.82	(0.31–2.12)	0.677
Diabetes mellitus	40	(20.6%)	5	(17.2%)	0.80	(0.29–2.23)	0.673
Hypertension	88	(45.4%)	13	(44.8%)	0.98	(0.45–2.15)	0.957
Dyslipidemia	67	(34.5%)	11	(37.9%)	1.16	(0.52–2.59)	0.721
Chronic kidney disease	14	(7.2%)	2	(6.9%)	0.95	(0.21–4.42)	0.950
Cardiovascular disease	22	(11.3%)	1	(3.4%)	0.28	(0.04–2.16)	0.221
Cirrhosis and hepatitis	33	(17.0%)	5	(17.2%)	1.02	(0.36–2.86)	0.975
Pulmonary diseases	8	(4.1%)	0	(0%)		-	-
Neurological disorders	19	(9.8%)	1	(3.4%)	0.33	(0.04–2.56)	0.288
Rheumatic diseases	13	(6.7%)	2	(6.9%)	1.03	(0.22–4.82)	0.969
Malignancy	15	(7.7%)	5	(17.2%)	2.49	(0.83–7.46)	0.104
FH of gastric cancer (%)	3	(1.5%)	0	(0%)		-	-
Smoking (%)	33	(17.6%)	4	(16.0%)	0.89	(0.29–2.76)	0.839
Alcohol (%)	47	(25.1%)	4	(16.0%)	0.57	(0.19–1.74)	0.321

**Table 5:** Multivariate analysis of associated risk factors and odds ratio in patients with complete and incomplete intestinal metaplasia.

Risk factors	Odds ratio (95% CI)		p-value
Male	1.16	(0.47–2.84)	0.749
Age >50 years	0.92	(0.27–3.12)	0.897
**BMI ≥23 kg/m**^**2**^	**3.25**	**(1.14–9.27)**	**0.027**

Patients with *H*. *pylori* infection were treated by sequential, concomitant, or triple therapy as suggested by GAT. *H*. *pylori* eradication rate, which was confirmed by urea breath test, was approximately 90%. Of 146 *H*. *pylori*-infected patients with IM, 78 underwent follow-up endoscopy and gastric biopsy. Fifty patients with IM who had successful *H*. *pylori* eradication underwent follow-up endoscopy and biopsy which demonstrated IM disappearance (62%) and IM persistence (38%) with the mean follow-up time of 21 months.

Of 37 patients with dysplasia, 26 (70.3%) had prior IM along with dysplasia on their gastric pathology and 27 (73%) had *H*. *pylori* infection. There were 12 patients having progression to dysplasia with the mean follow-up time of 23 months. Of 12 patients, 7 (58.3%) still had persistent *H*. *pylori* infection. During 20 months of study, there were 15 patients (1.1%) diagnosed with gastric cancer and 5 of them had history of prior IM along with adenocarcinoma. The 1-year survival rate of 15 gastric cancer patients was as low as 60%.

**Fig 4:**
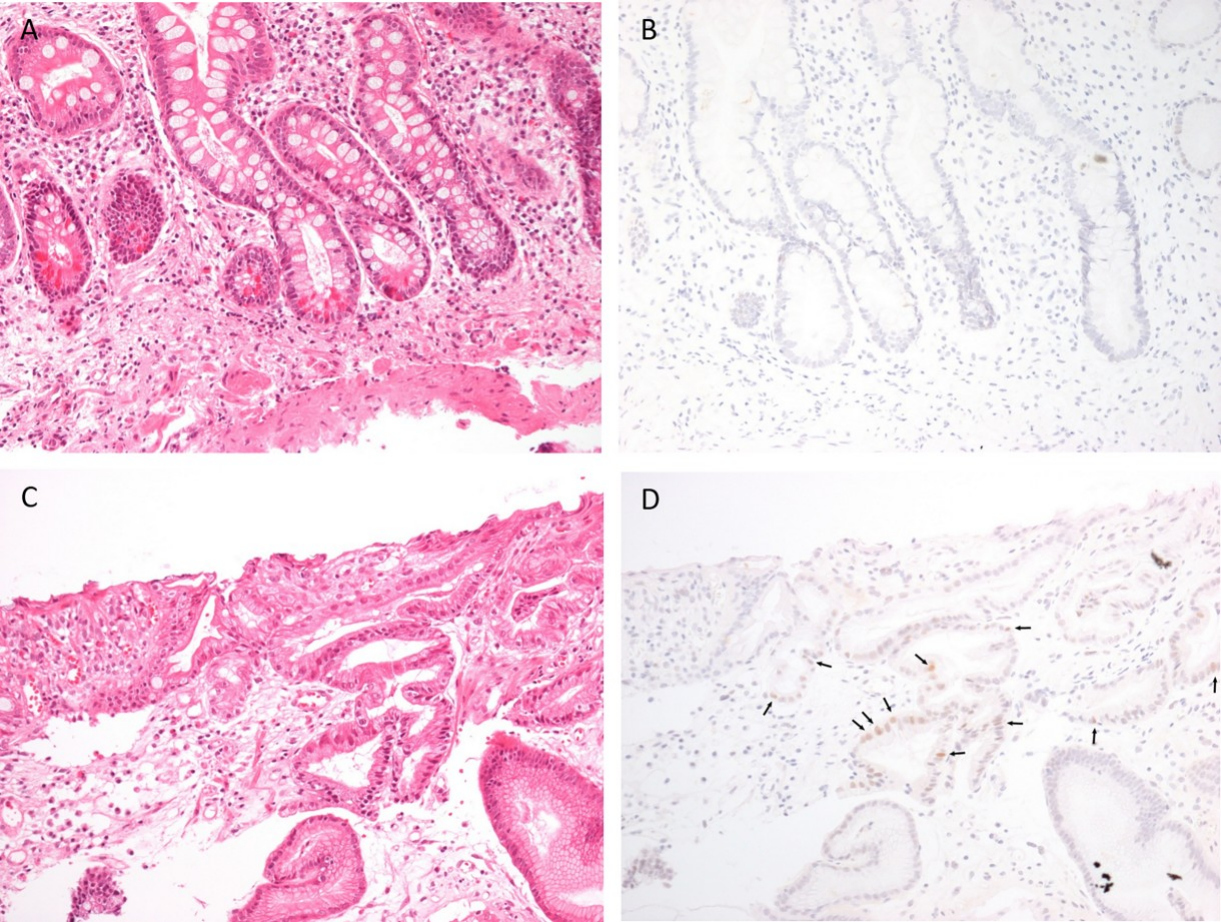
Histopathology of gastric intestinal metaplasia. (A) and (B) demonstrated complete IM, whereas (C) and (D) were incomplete IM. (A) and (C) were stained with hematoxylin and eosin (H&E). (B) demonstrated negative p53 staining, while (D) had positive p53 with brown nuclear staining of the epithelial cell (arrow).

Immunochemistry staining for p53 was performed in 107 available tissue samples with intestinal metaplasia (92 patients with complete IM and 15 with incomplete IM). Of 107 gastric tissue samples, only one showed positive result for p53 staining (Fig 4). The patient was a 58-year-old man diagnosed with incomplete IM on his gastric pathology. He denied smoking, alcohol drinking, or a family history of gastric cancer. The patient with positive p53 staining of IM had no difference of age (58 vs. 63.6 years, p = 0.630) and gender (male 1.9% vs. 0%, p = 0.495) from patients with negative p53.

## Discussion

Gastric cancer used to be responsible for the highest cancer death worldwide since 1930 but has been declining to the third rank after lung and colorectal cancer during the past 30 years [21]. *H*. *pylori* eradication and a decrease in salt-preserved food consumption are important factors in reducing the risk of developing gastric cancer [22]. Geographical variation in gastric cancer incidence was observed across regions around the world. Japan and the republic of Korea were among countries with the highest gastric cancer incidence, whereas the rate in the USA remained low. Thailand, despite being located in Asia, had low age-standardized gastric cancer incidence rate like the USA. Moreover, the trend over the forthcoming decades of these two countries is also predicted to be approximately the same [23]. The population in this study included only Thai people. Gastric intestinal metaplasia (IM), the primary focus of our study, had the prevalence of 16.3% which was higher than 7.5% from the large retrospective study in the United States [24]. This could be because Thailand has higher prevalence of *H*. *pylori* infection which is a notable associated risk factor for the development of IM [25, 26]. The nationwide study in the Netherlands revealed quite similar tendency that the proportion of gastric precancerous lesions increased with age and IM was the most common premalignant lesion [10]. The youngest patient in this study with gastric precancerous lesion was a 16-year-old female with *H*. *pylori*-associated atrophic gastritis. Although the prevalence of atrophic gastritis and IM in Thai patients younger than 30 years was relatively lower than the other study from Japan, it should be realized that precancerous lesions could be developed at an early age and *H*. *pylori* eradication is essential to prevent further progression of premalignant gastric lesions [25, 27].

Host and environmental factors influencing on the development of IM have been identified by several studies. This study demonstrated that males and age >50 years were both significantly associated with IM compared to the chronic gastritis group. Male predominance was also observed in the study in US veterans. The difference was that there were much higher numbers of male subjects than ours [28]. This could be explained by higher level of gene expression and inflammatory cytokines causing more severe gastric mucosal inflammation in *H*. *pylori*-infected males [29]. The prevalence of *H*. *pylori* infection in males with IM (68.9%) was not significantly different from females (61.5%) in our study. This study demonstrated that the development of IM was also related to age above 50 years, which was the same as the cut-off point to perform EGD in patients with alarm features in Thailand as a consequence of the doubling age-specific incidence rate of gastric cancer at this age [14]. The strongest modifiable risk factor for IM was current *H*. *pylori* infection, which could result in persistent gastric inflammation affecting epithelial differentiation and promoting malignant transformation [9].

IM can be further categorized into two types: complete and incomplete IM. The prevalence of incomplete IM (13%) in this study was lower than complete IM (87%). The prevalence of incomplete IM rose with increased age, peaked in the age group of 51–60 years and remained constant over 10% until the decline at very advanced age (>80 years). The previous study demonstrated that incomplete IM was associated with approximately three times higher rate of progression to gastric cancer than complete IM [30]. Therefore, the risk of having incomplete IM should be considered when patients enter middle age. Overweight and obese patients were at significant higher risk for the development of incomplete IM than complete IM. Overweight (BMI 23–24.9 kg/m^2^) and obesity (BMI ≥ 25 kg/m^2^) were defined by Asian BMI criteria [31]. The prior study established molecular mechanisms that obesity could potentiate proinflammatory cytokine production and promote gastric carcinogenesis in *Helicobacter*-infected mice [32]. Maintaining normal BMI might be beneficial as it is associated with reduced risk for the development of incomplete IM.

The p53 is a tumor suppressor gene involving in DNA repair, regulating cell cycle, maintaining genomic stability, and eventually inducing apoptosis. The p53 mutation was previously observed in 0–77% of gastric cancer [33]. This study showed only one positive p53 staining from the patient with incomplete IM. The earlier research revealed lower amount of p53 expression in IM (52%) than IM with concurrent gastric cancer (75%) [34]. This can be implied that p53 expression is present in the late stage of gastric precancerous cascade. While majority of patients in this study had complete IM, the p53 expression rate could be expected to be low. Our study also observed no significant association between p53 expression in patients with IM and demographic factors including gender and age.

The model of gastric precancerous cascade has been postulated by Correa for nearly 50 years. Recently, several guidelines have been developed on the management of premalignant gastric lesions to prevent and detect early gastric cancer. The endoscopic follow-up after successful *H*. *pylori* eradication in patients with IM predominantly demonstrated disappearance of IM on pathology. In addition, more than half of patients who had prior IM progression to dysplasia still had persistent *H*. *pylori* infection. Although IM has been considered as “the point of no return”, the result of this study emphasizes the importance of *H*. *pylori* eradication in order to prevent the progression of gastric precancerous lesions [15].

In conclusion, males, age >50 years, and current *H*. *pylori* infection are significant predictors for the presence of intestinal metaplasia. Body mass index might be beneficial for using as a predictive risk factor to reduce the development of incomplete intestinal metaplasia. *H*. *pylori* eradication could be an effective way to prevent the development of gastric precancerous lesions.

## Supporting information

S1 Table Laboratory results between complete and incomplete IM group (mean ± SD). (DOCX)
