# Supplementary material for: Predictors for development of complete and incomplete intestinal metaplasia (IM) associated with H. pylori infection: A large-scale study from low prevalence area of gastric cancer (IM-HP trial)
Source: PLoS One. 2020 Oct 1;15(10):e0239434. doi: 10.1371/journal.pone.0239434 (PMC7529201; doi:10.1371/journal.pone.0239434)
Supplement: S1 Table — (DOCX) [file pone.0239434.s001.docx]

**S1 Table.** Laboratory results between complete and incomplete IM group (mean ± SD)

| **Laboratory results** | **Complete IM** | **Incomplete IM** | **P-value** |
| --- | --- | --- | --- |
| Hemoglobin (g/dL) | 12.0 ± 2.2 | 11.6 ± 2.0 | 0.464 |
| WBC count (x10^9^/L) | 7.0 ± 2.5 | 7.8 ± 3.1 | 0.203 |
| Platelet count (x10^9^/L) | 237 ± 90 | 204 ± 61 | 0.115 |
| Plasma glucose (mg/dL) | 114 ± 31 | 109 ± 23 | 0.603 |
| Hemoglobin A1c (%) | 6.3 ± 1.0 | 6.3 ± 1.5 | 0.962 |
| CEA (ng/mL) | 4.8 ± 8.1 | 1.8 ± 1.0 | 0.638 |
| CA 19-9 (U/mL) | 49.2 ± 76.0 | 18.9 ± 12.1 | 0.618 |

WBC = White blood cell, CEA = Carcinoembryonic antigen, CA 19-9 = Carbohydrate antigen 19-9
